# Lung Ultrasound in Patients With SARS‐COV‐2 Pneumonia: Correlations With Chest Computed Tomography, Respiratory Impairment, and Inflammatory Cascade

**DOI:** 10.1002/jum.15831

**Published:** 2021-09-17

**Authors:** Gerardina Fratianni, Gabriella Malfatto, Elisa Perger, Luca Facchetti, Laura Pini, Miriam Bosco, Franco Cernigliaro, Giovanni B. Perego, Mario Facchini, Luigi P. Badano, Gianfranco Parati

**Affiliations:** ^1^ Department of Cardiology Istituto Auxologico Italiano IRCCS, Ospedale S. Luca Milan; ^2^ Dipartimento di Medicina e Chirurgia, Università di Milano‐Bicocca Milan; ^3^ Department of Radiology, ASST Spedali Civili di Brescia Brescia Italy; ^4^ Respiratory Medicine Unit, ASST Spedali Civili di Brescia Brescia Italy; ^5^ Department of Clinical and Experimental Sciences University of Brescia Brescia Italy

**Keywords:** chest CT, COVID‐19, cytokine cascade, lung ultrasound, pneumonia, SARS‐CoV‐2

## Abstract

**Objectives:**

Lung ultrasound (LUS) might be comparable to chest computed tomography (CT) in detecting parenchymal and pleural pathology, and in monitoring interstitial lung disease. We aimed to describe LUS characteristics of patients during the hospitalization for COVID‐19 pneumonia, and to compare the extent of lung involvement at LUS and chest‐CT with inflammatory response and the severity of respiration impairment.

**Methods:**

During a 2‐week period, we performed LUS and chest CT in hospitalized patients affected by COVID‐19 pneumonia. Dosages of high sensitivity C‐reactive protein (HS‐CRP), d‐dimer, and interleukin‐6 (IL‐6) were also obtained. The index of lung function (*P*/*F* ratio) was calculated from the blood gas test. LUS and CT scoring were assessed using previously validated scores.

**Results:**

Twenty‐six consecutive patients (3 women) underwent LUS 34 ± 14 days from the early symptoms. Among them, 21 underwent CT on the same day of LUS. A fair association was found between LUS and CT scores (*R* = 0.45, *P* = .049), which became stronger if the B‐lines score on LUS was not considered (*R* = 0.57, *P* = .024). LUS B‐lines score correlated with IL‐6 levels (*R* = 0.75, *P* = .011), and the number of involved lung segments detected by LUS correlated with the *P*/*F* ratio (*R* = 0.60, *P* = .019) but not with HS‐CRP and d‐Dimer levels. No correlations were found between CT scores and inflammations markers or *P*/*F*.

**Conclusion:**

In patients with COVID‐19 pneumonia, LUS was correlated with both the extent of the inflammatory response and the *P*/*F* ratio.

**Abbreviations**
CRP: C‐reactive protein
CT: computed tomography
ER: emergency department
HS‐CRP: high sensitivity C‐reactive protein
ICU: Intensive care unit
IL‐6: interleukin‐6
LUS: lung ultrasound

Lung ultrasound (LUS) examination is easy to learn compared to other imaging techniques. Moreover, it carries no risk of X‐ray exposure and can be performed at bedside with portable equipments.^1^ These advantages are among the reasons why LUS has gradually become a routine practice to assess lungs in many emergency departments (ER) and intensive care units, as an extension of bedside patient's examination.^2^, ^3^

LUS might be comparable to chest X‐ray and to chest computed tomography (CT) in detecting lung parenchyma and pleural pathology, and to monitor the response to treatment in interstitial lung disease.^4^, ^5^

During the recent severe acute respiratory syndrome coronavirus 2 (SARS‐CoV‐2) disease (COVID‐19), pandemic clinicians have looked for novel and noninvasive ways to safely evaluate patients suspected of COVID‐19 pneumonia.^6^, ^7^, ^8^ In the early phases of the disease, the chest CT scan of the patients may show relevant lung lesions before the onset of severe clinical symptoms.^9^, ^10^ However, in nonhospital settings, it is quite difficult to routinely implement CT for COVID‐19 symptomatic patients screening, due to logistic and safety reasons.

As observed during the 2013 avian influenza A (H7N9) epidemic,^11^, ^12^ LUS may aid in the identification and subsequent monitoring of suspected COVID‐19 pulmonary lesions.^13^, ^14^ Indeed, a number of case reports and small studies have emerged in the recent literature showing the clinical value of LUS in patients with confirmed COVID‐19 infections.^15^, ^16^, ^17^, ^18^, ^19^ Moreover, in mechanically ventilated patients due to COVID‐19 pneumonia hospitalized in Intensive care unit (ICU), LUS represented a potential indicator of respiratory function and inflammatory status.^20^

Accordingly, we designed a prospective, observational study to describe the information provided by this technique during the hospitalization due to COVID‐19 pneumonia, and to compare the relationships between LUS and chest‐CT scores with the levels of cytokine activation and the severity of respiratory impairment.

## Materials and Methods

### Subjects

This is a prospective, observational single‐center study, carried out at San Luca Hospital, a clinical research Institution in Milan (Italy) fully turned into a dedicated COVID‐19 Unit at the time of the SARS‐CoV‐2 virus outbreak that severely hit Northern Italy between March and May 2020. In the frame of health care management in our region during the COVID‐19 outbreak, our hospital was assigned to receive patients from the ER of other hospitals, which transferred COVID‐19 patients to our unit after a first brief hospitalization. Due to the health care resource shortage related to the unpredictable progression of the pandemic, our Ethics Committee allowed us to include all patients consecutively hospitalized due to COVID‐19 pneumonia in our Unit over a 2‐week period, from April 18th to May 3rd, 2020. Within this time window, we performed LUS and chest CT in all patients admitted to our unit who had a laboratory‐confirmed SARS‐CoV‐2 pneumonia diagnosis, and who accepted to participate in the study. Given the above‐mentioned coordination among Lombardy hospitals, all patients were included during the hospitalization in our COVID‐19 Unit with a range of 4 to 28 days after the first ER evaluation, which took place in other hospitals of our city. Demographic and clinical data such as age, sex, weight and height, comorbidities, hospitalization information, and outcomes were collected. The study conformed to the standards set by the Declaration of Helsinki, and, as already mentioned, received ethical clearance from the Ethic Committee of Istituto Auxologico Italiano. All patients provided informed consent for the collection of their hospital clinical data for research purposes.

#### LUS Measurements and Scoring

LUS was performed using a previously described 16‐segment method.^3^, ^19^ The GE Healthcare Venue 40 ultrasound system (GE, USA) equipped with both a convex array ultrasound probe (2–2.5 MHz) and a high‐frequency linear array probe (5–13 MHz) was used in this study. The linear transducer was preferred to define the LUS score. The ultrasound probe and cable were kept in a sterile, plastic wrap, and were sterilized again after each study. The physician (operator) entered the patient's room respecting all the preventive measures for respiratory, droplet, and contact isolation provided by the World Health Organization for the SARS‐CoV‐2 outbreak. Images were recorded and digitally stored for further analysis. A normal LUS scan demonstrated only A‐lines that are a repetition of the pleural line at twice the distance from skin to the pleural line. These lines are indicative of air below the pleural line, corresponding to the parietal pleura. The A‐lines may be complete or broken. The B lines are described as hyper‐echogenic artifacts that resemble a “comet tail”. They arise from the pleural line and move in concert with a sliding lung. The B1 lines are associated with an interstitial syndrome and diminished lung aeration. The B2 lines are confluent lines appearing as a “white lung” (called also glass‐rockets), equivalent to CT ground‐glass opacities. The latter suggests a more severe loss of lung aeration. Lung consolidations (C) are associated with hepatization of lung parenchyma with or without air bronchograms, and suggest major loss of lung aeration (atelectasis versus pneumonia). As yet, there are no ultrasound appearances that would be pathognomonic of SARS‐CoV‐2‐related pneumonia. We used a standardized scanning scheme and scoring system already used for other forms of interstitial lung disease,^3^, ^21^ dividing the lungs into 16 segments and assigning to each segment a score as it follows: Score 0: predominant A‐lines or <3 separate B‐lines; Score 1: at least 3 B‐lines or coalescent B‐lines occupying ≤50% of the screen without a clearly irregular pleural line; Score 1p: at least 3 B‐lines or coalescent B‐lines occupying ≤50% of the screen with a clearly irregular pleural line; Score 2: coalescent B‐lines occupying >50% of the screen without a clearly irregular pleural line; Score 2p: coalescent B‐lines occupying >50% of the screen with a clearly irregular pleural line; and Score 3: large consolidations (at least 1 cm), further described as hypo‐echogenic, tissue‐like, with air bronchograms, etc. The presence of pleural effusion was also reported.

Finally, for each examination, we counted the number of segments involved irrespectively of their individual score, and we calculated three scores:

1. the total score (sum of the scores of each segment);
2. the “B‐lines” score (sum of scores 1 to 2p) not taking into account the consolidations; and
3. the “p” score, that is, the score of segments where B lines and irregular pleural lines were observed;

In this analysis, a score = 0 would reflect a normal lung, whereas a score = 48 would represent a severely damaged lung, where ubiquitous consolidation is present. LUS was performed by a clinician specialized in LUS, blind to laboratory test, or hospitalization outcomes. This scoring system has previously been validated.^21^

#### Chest CT

Chest CT scans were performed with the patient in the supine position and during inspiratory apnea using GE LightSpeed (GE Healthcare, Milwaukee, WT). The gantry was maintained without any inclination, and no iodinated contrast medium was used. Chest CT scoring was performed by a radiologist blinded for patients LUS score or hospitalization outcomes, using validated score.^22^, ^23^ Briefly, each lung was divided into three zones: upper (above the carina), middle, and lower (below the inferior pulmonary vein) zones; each zone was evaluated for percentage of lung involvement on a scale of 0 to 4 (0, 0% involvement; 1, less than 25% involvement; 2, 25% to less than 50% involvement; 3, 50% to less than 75%; 4, 75% or greater involvement). Overall CT scores were the summation of scores from all six lung zones. The maximum possible score was 25 (24 + 1 in case of consolidations).^22^, ^23^

#### Blood Tests, Arterial Gas‐Analysis, and Respiratory Impairment

Interleukin‐6 (IL‐6), high‐sensitivity C‐reactive protein (CRP), d‐Dimer, and fibrinogen, among other tests, were routinely determined in patients every 24 hours to check for disease evolution and response to treatment during hospitalization. In particular, IL‐6 was determined with a high sensitivity system (Elecsys® IL‐6 test, Roche). Arterial gas‐analysis was also obtained every day from an arterial line. To assess lung function, the ratio between the arterial oxygen pressure (PaO_2_) and the inspired fraction of oxygen (FiO_2_) (*P*/*F*) was calculated, and a value <200 was considered an index of severe respiratory distress.

#### Statistical Analysis

Data were summarized with numbers and percentages, mean ± standard deviation. Continuous variables were compared using a two‐tailed unpaired *t*‐test or, for asymmetrically distributed data, the Mann–Whitney test, as appropriate after testing for normality with Shapiro–Wilk's test. Fisher's exact test was used to compare categorical variables. Due to the high dispersion of the results, IL‐6 log10 value was used in the analysis. Correlations among variables were evaluated with Spearman's correlation test (*r*). A *P*‐value of <.05 was considered statistically significant. Statistical analyses were performed using OriginPro (OriginPro 7.0, Microcal, USA).

## Results

A total of 26 patients were included in the study and performed LUS. None of them were receiving mechanical ventilation. Table 1 shows the main demographic and clinical characteristics of this study group. Twenty‐one patients also had chest CT scan within 24 hours from LUS. Due to their clinical history of co‐existence of worsening of heart failure, confirmed by cardiac echocardiography, and blood B‐type natriuretic peptide levels, three patients (age 43, 89, and 72 years, all males) were excluded from the final cohort, leaving 23 patients who underwent LUS, and 18 who received both LUS and chest CT. Five patients could not perform CT scan for technical problems and were not included in the comparative analyses. LUS was performed 34 ± 14 days from symptoms onset (range, 7–48 days). The latter was defined by the first COVID‐19 symptom reported before hospitalization and ER evaluation. In two patients, the LUS was repeated 1 week after the first test for clinical reasons. The first patient (male, 74 years old) showed a rapidly worsening clinical status and eventually died. The second one (male, 89 years old) slowly recovered, and he was discharged alive and well after two more weeks. The time from symptoms onset to the echo test did not relate either with the type or severity of the lesions observed with LUS or with CT scan.

**TABLE 1:** Clinical Characteristics of the Patients With COVID‐19 Pneumonia Who Performed Lung Ultrasound

Number	26
Males/females	23/3
Age (y)	66 ± 15
Diabetes/hypertension/cardiovascular disease	5/20/14
>1 risk factor	22
≥ 2 risk factors	16
Days from illness onset to LUS and CT	34 ± 14
Signs and symptoms (%)	
Fever	100
Dyspnea	100
Cough	80
Fatigue	80
Laboratory findings	
Lymphocytes count (10^−9^/L)	1.07 ± 0.54
d‐dimer	2988 ± 1956
CRP	7.8 ± 5.6
IL‐6	617 ± 157
Outcome	
Death	3
Discharged home alive	23

CRP indicates C‐reactive protein; CT, computed tomography; IL‐6, Interleukin‐6; LUS, lung ultrasound.

We found LUS abnormalities all over the lung parenchyma in our patients. However, there was a tendency of the lesions to prevail in the posterior‐basal areas of both lungs, and to be slightly more evident in the right lung (221 of 368 affected lung segments, of them 60% being in the right posterior‐basal lobe). We examined 368 lung segments, and we found many different types of lesions (some of them coexisting in the same patient): rough and discontinuous pleural lines (165/368 segments), subpleural consolidation (100/368 segments), air bronchogram sign or air bronchiologram sign in subpleural consolidation (20/368 segments), visible B lines (195/368 segments), localized pleural thickening (55/368 segments), and localized pleural effusion (19/368 segments).

**FIGURE 1:**
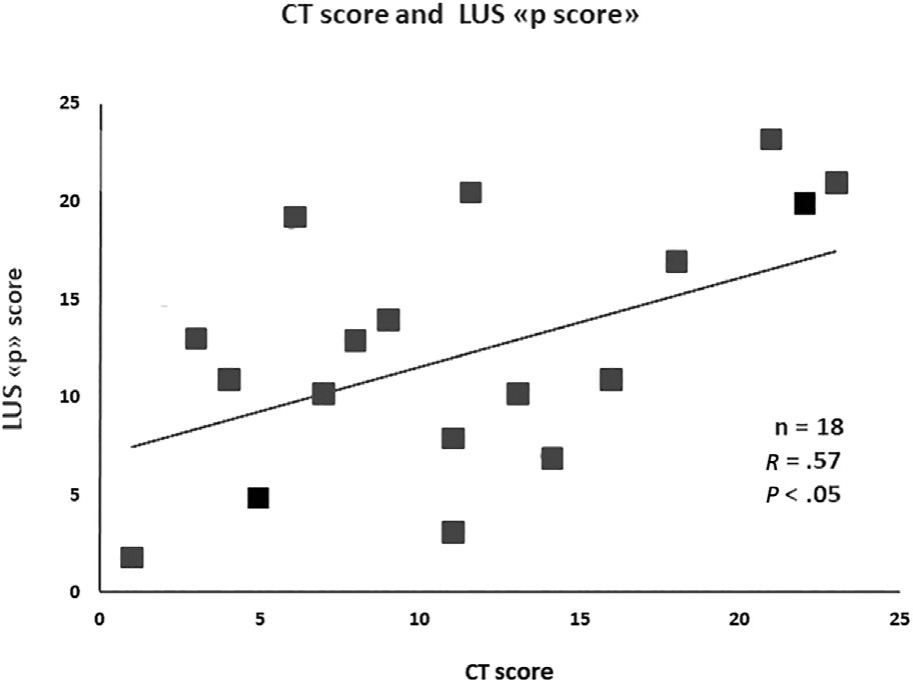
Correlation between computed tomography score and lung ultrasound score, including pleural abnormalities, that is, the “p” score.

The correlation between total LUS and chest CT scores was weak (*r* = 0.45, *P* = .049). However, it increased when CT score was compared with the “p” score only (i.e. when B‐lines *and* subpleural consolidations were both present) (*r* = 0.57, *P* = .024) (Figure 1).

**FIGURE 2:**
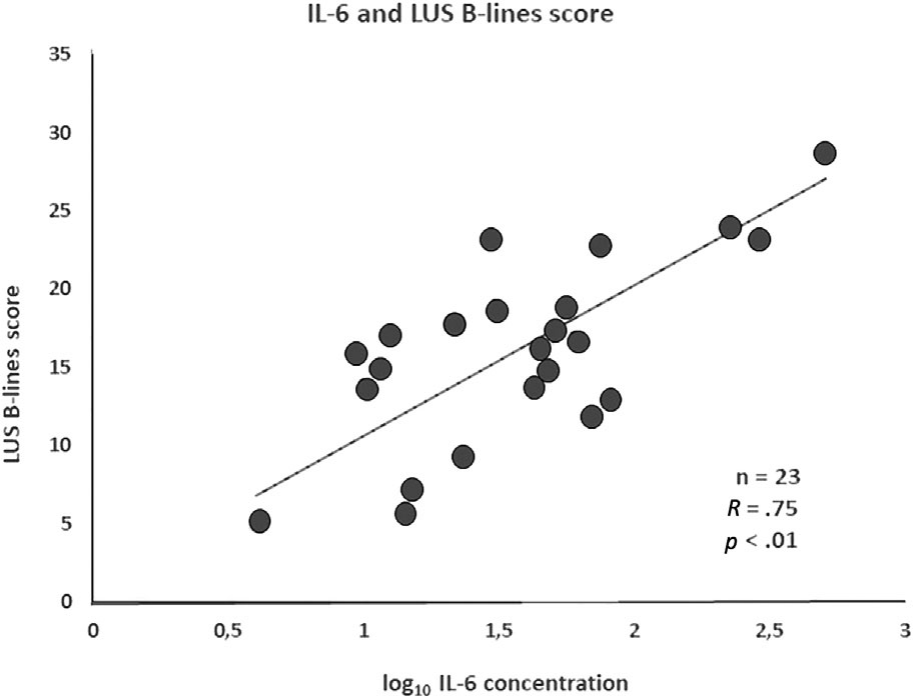
Correlation between B‐lines score and interleukin‐6 blood levels.

No relationship was observed between CT score and any of the inflammation markers (IL‐6, fibrinogen, and HS‐CRP levels). Conversely, the IL‐6 levels were positively and significantly correlated with the B‐lines score obtained with LUS (*r* = 0.75, *P* = .011) (Figure 2).

**FIGURE 3:**
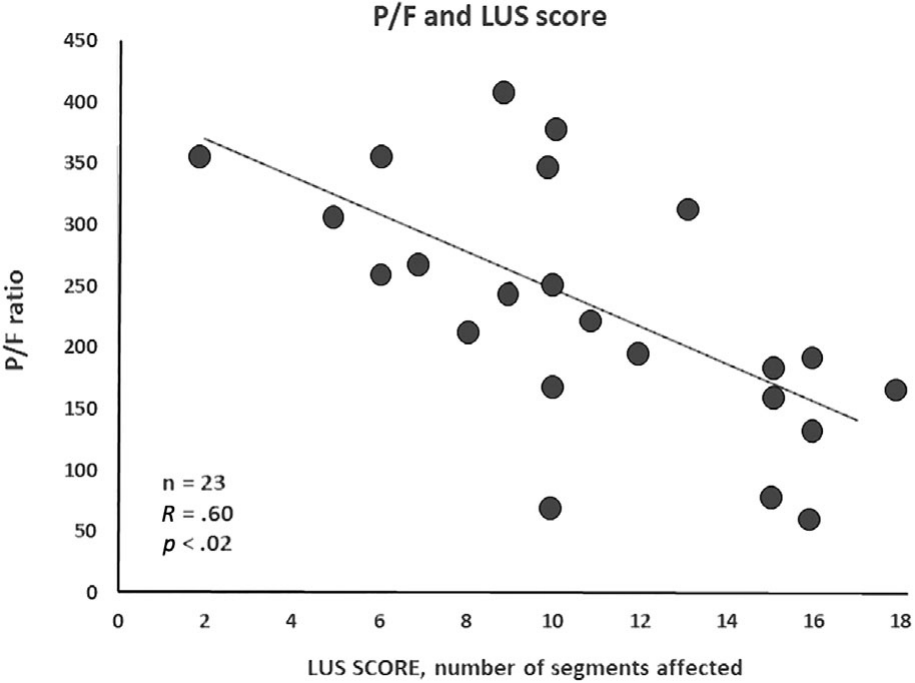
Correlation between total lung segments involved in lung ultrasound analysis and *P*/*F* at blood gas tests.

Patients with severe respiratory impairment (*P*/*F* ratio less than 200) who were older than patients with *P*/*F* ratio >200 showed a lower number of lymphocytes in the venous blood, and the levels of d‐dimer and IL‐6 were significantly higher than in those with less‐severe disease. Moreover, patients with *P*/*F* ratio <200 showed higher LUS and CT scores (Table 2). A negative correlation was observed between *P*/*F* values and the number of abnormal lung segments on LUS (*r* = 0.60, *P* = .019) (Figure 3). Whereas, no relationship was observed between *P*/*F* values and CT score (*r* = −0.16, *P* = .52), or the number of abnormal lung segments on CT scan (*r* = −0.93, *P* = .12).

**TABLE 2:** Demographic and Clinical Differences Between Patients According to the Severity of Their Respiratory Conditions

	All	*P*/*F* ≥200	*P*/*F* <200
Number	23	13	10
Males/females	20/3	11/2	9/1
Age (y)	66 ± 14	62 ± 15	71 ± 14
Diabetes/hypertension/cardiovascular disease	7/21/11	2/11/7	5/10/4
>1 risk factor	21	10	11
≥2 risk factors	17	8	9
Days from illness onset to LUS	34 ± 14	29 ± 15	35 ± 16
*P*/*F* value	236 ± 106	280 ± 63	159 ± 84*
Global LUS score	17.5 ± 7.6	13.3 ± 5.2	23.7 ± 7.1*
LUS B‐lines score	15.4 ± 6.6	12.6 ± 5.1	20.5 ± 5.9*
LUS p‐score	12.6 ± 6.8	9.7 ± 5.6	17.5 ± 6*
LUS segments involved	10.5 ± 4.1	8.4 ± 2.9	14.3 ± 2.8*
Global CT score	12.1 ± 6.3	10.2 ± 7.1	13.3 ± 6.0*
CT segments involved	5.2 ± 1.5	4.7 ± 1.8	6.0 ± 0
Signs and symptoms (%)			
Fever	100	100	100
Dyspnea	100	100	100
Cough	70	62	80
Fatigue	74	69	80
Laboratory findings			
Lymphocytes count (10^−9^/L)	0.98 ± 0.59	1.15 ± 0.59	0.88 ± 0.60*
d‐dimer	2933 ± 3634	1931 ± 1245	4223 ± 2935*
HS‐CRP	8.4 ± 6.1	7.5 ± 5.1	9.2 ± 7.0
IL‐6	75 ± 118	27 ± 18	124 ± 117*
Outcome			
Death	3	0	3
Discharged	20	13	7

CT indicates computed tomography; HS‐CRP, high sensitivity C‐reactive protein; IL‐6, interleukin‐6; LUS, lung ultrasound; P/F, ratio between the arterial oxygen pressure and the inspired fraction of oxygen.

* *P* < .05 between groups.

In the two patients in whom LUS was repeated after 1 week, there was a divergent evolution of the lung abnormalities. In the patient who died a few days after being tested, HS‐CRP was 2.57 mg/dl in the first evaluation and decreased to 0.56 mg/dl, the total LUS score increased from 21 to 26, and the B‐lines score increased from 18 to 23. Moreover, the number of lung segments involved increased (from 11 to 16) and the sub‐pleural consolidations became visible in all 16 segments. Conversely, in the patient who improved and was eventually discharged at home, HS‐CRP decreased from 11.4 to 0.93 mg\dl after 7 days, the total LUS score decreased from 37 to 28, the B‐lines score decreased from 28 to 16, and the number of lung segments involved remained stable (14 and 13). However, there was a remarkable reduction of the extent of the sub‐pleural consolidations (being present in all 16 lung segments at first examination and in only 5 segments in the second test). Unfortunately, CT scan was not performed in these two patients during the follow‐up.

## Discussion

Our data show that LUS, performed during the hospitalization for COVID‐19 pneumonia, can detect the occurrence of lung damage, and provide semi‐quantitative indices related to clinical markers of disease severity. In particular, we show that LUS can clearly identify signs of interstitial‐alveolar damage in patients with mild‐to‐severe COVID‐19 pneumonia. These signs include diffuse pleural line abnormalities, sub‐pleural consolidations, white lung areas, and thick, irregular vertical abnormalities. We also show that these LUS abnormalities were associated with clinical response. Indeed, while some LUS patterns (such as B‐lines presence) were strictly related to laboratory markers of cytokine's pathway activation, in particular, with an increase in IL‐6, other LUS abnormalities (i.e. the number of damaged lung segments) seemed to be associated with the severity of respiratory impairment.

Our findings provide additional evidence in support of the increasing use of LUS in the emergency and intensive care settings for the bedside evaluation of critically ill patients.^2^, ^3^, ^4^ Noticeably, use of this approach in the evaluation of suspected or confirmed SARS‐CoV‐2 patients (and in general during high‐risk infectious disease outbreaks) offers several potential advantages.^13^, ^14^, ^20^ Lung images can be obtained at bedside, while the use of traditional imaging such as chest CT scan require the patient to be moved to the radiology unit with the potential exposure of several people to the risk of infection.^21^ Regular repetitions of LUS may add relevant information on the degree of lung tissue involvement in the clinical course of the disease, helping in treatment decisions and limiting the need for chest X‐ray and CT scan repetition,^24^ at the same time providing prognostic markers, as shown in our two patients who had LUS repeated during their hospital stay and in a recent case report.^25^

The three complementary LUS scores used in our study highlighted different pathophysiologic aspects of COVID pneumonia. The global score unquestionably reflected the global lung damage. On the other hand, the B‐lines score might assess the extension of the interstitial damage before the lung consolidation takes place. Indeed, in our cohort, this score was significantly related to the levels of IL‐6, whose high levels are thought to reflect the inflammatory state of the lungs.^26^, ^27^ Thus, high density of B‐lines evaluated by LUS in association with increasing levels of IL‐6 may represent an early marker of an ongoing interstitial damage consequent to a severe inflammatory process affecting the lungs. Consisting with our results, in mechanical ventilated subjects in the ICU, LUS score correlated with gas exchange and IL‐6 plasma concentration.^20^ Recently, in a multicenter study, LUS represented a strong independent predictor of CRP positivity (odds ratio 4.2, confidence interval 2.6–6.7, *P* < .0001).^28^ Early identification of the inflammatory cascade activation has been shown to have implications for clinical decisions, such as the use of Tocilizumab or similar IL modulators^29^, ^30^, ^31^ and dexamethasone.^32^ On the other hand, independently of the severity of the lesions observed, the number of lung segments involved was significantly higher in patients showing severe acute respiratory distress syndrome, suggesting that also the degree of lung infection diffusion may be a threat to patients' life. Despite the small sample size, consistently with Bonadia et al, we also showed that a larger proportion of lung area involved by COVID‐19 pneumonia assessed by LUS was typical of patients with worst prognosis.^33^

### Comparison LUS and Chest CT

It has already been stated that LUS is a highly sensitive technique. In a recent report by Lieveld et al, comparing LUS and CT scan for the diagnosis of COVID‐19 pneumonia, sensitivity and specificity for LUS were 91.9% and 71.0% versus 88.4% and 82.0% for CT, respectively,^34^ making them almost superimposable for COVID‐19 diagnosis.

As a matter of fact, some patterns at LUS are indicative of pulmonary alteration, but are not disease‐specific.^21^ As an example, the presence of B‐lines is reported in several other pathologies in addition to COVID‐19, such as pulmonary edema, interstitial fibrosis, and asthma.^35^ Indeed, when the presence of B lines was associated with pleural abnormalities, and only the “p” LUS score was taken into account, the relationship between the two methods became more evident.

The aim of this report, however, was not a comparison of the two techniques for the diagnosis of COVID‐19 pneumonia. Due to the health care organization requirements in our region, our patients were studied about 1 month from the beginning of their symptoms, and they were still seriously ill. In such a condition, LUS and CT scans were performed as an aid to the clinical follow‐up and therapeutic decisions. In this specific and yet undescribed clinical setting, the LUS and CT scores were both related to the severity of respiratory impairment, as recently shown in a similar study in artificially ventilated patients.^36^ However, LUS seemed superior to CT in the detection of interstitial abnormalities linked to cytokine activation,^37^ while CT scan‐related scores would not easily detect these inflammatory‐related interstitial fluid changes. Characterized by more accurate tissue density resolution, LUS is highly sensitive to changes in lungs balance between air and fluids. COVID‐19 pneumonia is known to be characterized by alveolar‐interstitial damage with inflammatory exudation and edema, which can be detected as B‐lines by LUS. Differently, CT scan might miss early detection of inflammation‐related lung water changes. Our results showing a better correlation of inflammatory markers with LUS rather than with CT support this hypothesis. Indeed, our findings are in line with the already reported higher sensitivity of LUS with respect to CT.^34^, ^38^

Another peculiar and favorable feature of our study is the evaluation of COVID‐19 patients during the hospitalization but after the first evaluation in the ER, which has allowed us to highlight the importance of LUS in the evaluation of lung lesions during the hospitalization.

### Limitations

A few limitations of our study have to be acknowledged. First, the sample size of our study is relatively small. This limited size was determined by the emergency conditions in our region in which we were operating during this pandemic. This unavoidable limitation, has, however, also offered us the possibility to perform a real‐life evaluation of hospitalized patients selectively in a post‐acute stage of COVID‐19 pneumonia over a 2 weeks' period. In spite of the difficult conditions under which our data were collected due to the health emergency related to COVID‐19 outbreak in Northern Italy last Spring, we have nevertheless been able to provide the diagnostic value of LUS, and of the clinical relevance of the indices it can yield.

Given that our results were selectively derived from patients during the hospitalization, our results are not generalizable to all stages of COVID‐19 pneumonia.

## Conclusions

LUS may serve as an easy‐to‐use bedside tool to improve the evaluation of lung involvement in COVID‐19 pneumonia. It is a valuable alternative to lung CT and it may help in easily following the evolution of the disease. Our findings should stimulate their confirmation in larger groups of patients.

## References

[1] Anderson KL , Fields JM , Panebianco NL , Jenq KY , Marin J , Dean AJ . Inter‐rater reliability of quantifying pleural B‐lines using multiple counting methods. J Ultrasound Med 2013; 32:115–120. 10.7863/jum.2013.32.1.115. 23269716

[2] Narula J , Chandrashekhar Y , Braunwald E . Time to add a fifth pillar to bedside physical examination: inspection, palpation, percussion, auscultation, and Insonation. JAMA Cardiol 2018; 3:346–350. 10.1001/jamacardio.2018.0001. 29490335

[3] Bouhemad B , Mongodi S , Via G , Rouquette I . Ultrasound for "lung monitoring" of ventilated patients. Anesthesiology 2015; 122:437–447. 10.1097/aln.0000000000000558. 25501898

[4] Zhao Z , Jiang L , Xi X , et al. Prognostic value of extravascular lung water assessed with lung ultrasound score by chest sonography in patients with acute respiratory distress syndrome. BMC Pulm Med 2015; 15:98–98. 10.1186/s12890-015-0091-2. 26298866 PMC4546293

[5] Man MA , Dantes E , Domokos Hancu B , et al. Correlation between transthoracic lung ultrasound score and HRCT features in patients with interstitial lung diseases. J Clin Med 2019; 8:1119. 10.3390/jcm8081199. PMC6722523 31405211

[6] Smith AA , Fridling J , Ibrahim D , Porter PS Jr . Identifying patients at greatest risk of mortality due to COVID‐19: a New England perspective. West J Emerg Med 2020; 21:785–789. 10.5811/westjem.2020.6.47957. 32726242 PMC7390549

[7] Perger E , Soranna D , Pengo M , Meriggi P , Lombardi C , Parati G . Sleep‐disordered breathing among hospitalized patients with COVID‐19. Am J Respir Crit Care Med 2021; 203:239–241. 10.1164/rccm.202010-3886LE. 33180549 PMC7874403

[8] Messineo L , Perger E , Corda L , et al. Breath‐holding as a novel approach to risk stratification in COVID‐19. Crit Care 2021; 25:208. 10.1186/s13054-021-03630-5. 34127052 PMC8200551

[9] Xiong Y , Sun D , Liu Y , et al. Clinical and high‐resolution CT features of the COVID‐19 infection: comparison of the initial and follow‐up changes. Invest Radiol 2020; 55:332–339. 10.1097/rli.0000000000000674. 32134800 PMC7147282

[10] Li M , Lei P , Zeng B , et al. Coronavirus disease (COVID‐19): Spectrum of CT findings and temporal progression of the disease. Acad Radiol 2020; 27:603–608. 10.1016/j.acra.2020.03.003. 32204987 PMC7156150

[11] Tsai NW , Ngai CW , Mok KL , Tsung JW . Lung ultrasound imaging in avian influenza a (H7N9) respiratory failure. Crit Ultrasound J 2014; 6:6. 10.1186/2036-7902-6-6. 24949191 PMC4051407

[12] Zhang Y‐K , Li J , Yang J‐P , Zhan Y , Chen J . Lung ultrasonography for the diagnosis of 11 patients with acute respiratory distress syndrome due to bird flu H7N9 infection. Virol J 2015; 12:176–176. 10.1186/s12985-015-0406-1. 26503117 PMC4623238

[13] Soldati G , Smargiassi A , Inchingolo R , et al. Is there a role for lung ultrasound during the COVID‐19 pandemic? J Ultrasound Med 2020; 39:1459–1462. 10.1002/jum.15284. 32198775 PMC7228238

[14] Gaspardone C , Meloni C , Preda A , et al. Lung ultrasound in COVID‐19 a role beyond the acute phase? J Ultrasound Med 2021; 40:503–511. 10.1002/jum.15425. 32770687

[15] Vetrugno L , Bove T , Orso D , et al. Our Italian experience using lung ultrasound for identification, grading and serial follow‐up of severity of lung involvement for management of patients with COVID‐19. Echocardiography 2020; 37:625–627. 10.1111/echo.14664. 32239532 PMC7228311

[16] Buonsenso D , Piano A , Raffaelli F , Bonadia N , de Gaetano DK , Franceschi F . Point‐of‐care lung ultrasound findings in novel coronavirus disease‐19 pnemoniae: a case report and potential applications during COVID‐19 outbreak. Eur Rev Med Pharmacol Sci 2020; 24:2776–2780. 10.26355/eurrev_202003_20549. 32196627

[17] Convissar DL , Gibson LE , Berra L , Bittner EA , Chang MG . Application of lung ultrasound during the COVID‐19 pandemic: a narrative review. Anesthesia Analg 2020; 131:345–350. 10.1213/ane.0000000000004929. PMC7202122 32366774

[18] Thomas A , Haljan G , Mitra A . Lung ultrasound findings in a 64‐year‐old woman with COVID‐19. Can Med Assoc J 2020; 192:E399. 10.1503/cmaj.200414. 32234724 PMC7162441

[19] Peng QY , Wang XT , Zhang LN . Findings of lung ultrasonography of novel corona virus pneumonia during the 2019‐2020 epidemic. Intensive Care Med 2020; 46:849–850. 10.1007/s00134-020-05996-6. 32166346 PMC7080149

[20] Rojatti M , Regli IB , Zanforlin A , et al. Lung ultrasound and respiratory pathophysiology in mechanically ventilated COVID‐19 patients‐an observational trial. SN Compr Clin Med 2020; 1–8. 10.1007/s42399-020-00536-1. PMC7516234 32995708

[21] Gargani L , Soliman‐Aboumarie H , Volpicelli G , Corradi F , Pastore MC , Cameli M . Why, when, and how to use lung ultrasound during the COVID‐19 pandemic: enthusiasm and caution. Eur Heart J Cardiovasc Imaging 2020; 21:941–948. 10.1093/ehjci/jeaa163. 32515793 PMC7314093

[22] Ooi GC , Khong PL , Müller NL , et al. Severe acute respiratory syndrome: temporal lung changes at thin‐section CT in 30 patients. Radiology 2004; 230:836–844. 10.1148/radiol.2303030853. 14990845

[23] Wang Y , Dong C , Hu Y , et al. Temporal changes of CT findings in 90 patients with COVID‐19 pneumonia: a longitudinal study. Radiology 2020; 296:E55–e64. 10.1148/radiol.2020200843. 32191587 PMC7233482

[24] Peng Q‐Y , Wang X‐T , Zhang L‐N . Chinese critical care ultrasound study G. using echocardiography to guide the treatment of novel coronavirus pneumonia. Crit Care 2020; 24:143. 10.1186/s13054-020-02856-z. 32276598 PMC7146071

[25] Zieleskiewicz L , Duclos G , Dransart‐Rayé O , Nowobilski N , Bouhemad B . Ultrasound findings in patients with COVID‐19 pneumonia in early and late stages: two case‐reports. Anaesthesia Crit Care Pain Med 2020; 39:571–573. 10.1016/j.accpm.2020.05.016. PMC7333621 32654908

[26] Desai TR , Leeper NJ , Hynes KL , Gewertz BL . Interleukin‐6 causes endothelial barrier dysfunction via the protein kinase C pathway. J Surg Res 2002; 104:118–123. 10.1006/jsre.2002.6415. 12020130

[27] McElvaney OJ , McEvoy NL , McElvaney OF , Carroll TP , Murphy MP , Dunlea DM , Ní Choileáin O , Clarke J , O'Connor E , Hogan G , Ryan D Characterization of the inflammatory response to severe COVID‐19 illness. Am J Respir Crit Care Med 2020;202:812–821. 10.1164/rccm.202005-1583OC. 32584597 PMC7491404

[28] Volpicelli G , Gargani L , Perlini S , et al. Lung ultrasound for the early diagnosis of COVID‐19 pneumonia: an international multicenter study. Intensive Care Med 2021; 47:444–454. 10.1007/s00134-021-06373-7. 33743018 PMC7980130

[29] Jordan SC , Zakowski P , Tran HP , et al. Compassionate use of tocilizumab for treatment of SARS‐CoV‐2 pneumonia. Clin Infect Dis 2020; 71:3168–3173. 10.1093/cid/ciaa812. 32575124 PMC7337689

[30] Quartuccio L , Sonaglia A , McGonagle D , et al. Profiling COVID‐19 pneumonia progressing into the cytokine storm syndrome: results from a single Italian Centre study on tocilizumab versus standard of care. J Clin Virol 2020; 129:104444. 10.1016/j.jcv.2020.104444. 32570043 PMC7227535

[31] Huang E , Jordan SC . Tocilizumab for Covid‐19 ‐ the ongoing search for effective therapies. N Engl J Med 2020; 383:2387–2388. 10.1056/NEJMe2032071. 33296566 PMC7745168

[32] Noreen S , Maqbool I , Madni A . Dexamethasone: therapeutic potential, risks, and future projection during COVID‐19 pandemic. Eur J Pharmacol 2021; 894:173854. 10.1016/j.ejphar.2021.173854. 33428898 PMC7836247

[33] Bonadia N , Carnicelli A , Piano A , et al. Lung ultrasound findings are associated with mortality and need for intensive care admission in COVID‐19 patients evaluated in the emergency department. Ultrasound Med Biol 2020; 46:2927–2937. 10.1016/j.ultrasmedbio.2020.07.005. 32798003 PMC7362856

[34] Lieveld AWE , Kok B , Schuit FH , et al. Diagnosing COVID‐19 pneumonia in a pandemic setting: lung ultrasound versus CT (LUVCT) a multi‐Centre, prospective, observational study. ERJ Open Res 2020; 6:00539. 10.1183/23120541.00539-2020. 33442553 PMC7569754

[35] Staub LJ , Mazzali Biscaro RR , Kaszubowski E , Maurici R . Lung ultrasound for the emergency diagnosis of pneumonia, acute heart failure, and exacerbations of chronic obstructive pulmonary disease/asthma in adults: a systematic review and meta‐analysis. J Emerg Med 2019; 56:53–69. 10.1016/j.jemermed.2018.09.009. 30314929

[36] Zieleskiewicz L , Markarian T , Lopez A , et al. Comparative study of lung ultrasound and chest computed tomography scan in the assessment of severity of confirmed COVID‐19 pneumonia. Intensive care medicine 2020; 46:1707–1713. 10.1007/s00134-020-06186-0. 32728966 PMC7388119

[37] Gurkan OU , He C , Zielinski R , et al. Interleukin‐6 mediates pulmonary vascular permeability in a two‐hit model of ventilator‐associated lung injury. Exp Lung Res 2011; 37:575–584. 10.3109/01902148.2011.620680. 22044313 PMC3406407

[38] Yang Y , Huang Y , Gao F , Yuan L , Wang Z . Lung ultrasonography versus chest CT in COVID‐19 pneumonia: a two‐centered retrospective comparison study from China. Intensive Care Medicine 2020; 46:1761–1763. 32451581 10.1007/s00134-020-06096-1 PMC7246293

